# Associations of anemia with death and major bleeding in patients with atrial fibrillation: A report from the Chinese Atrial Fibrillation Registry Study

**DOI:** 10.1002/clc.23764

**Published:** 2021-12-28

**Authors:** Zhuxin Zhang, Chao Jiang, Liu He, Yu Bai, Jiahui Wu, Rong Hu, Qiang Lv, Man Ning, Li Feng, Ribo Tang, Caihua Sang, Deyong Long, Jianzeng Dong, Xin Du, Gregory Y. H. Lip, Changsheng Ma

**Affiliations:** ^1^ Department of Cardiology, Beijing Anzhen Hospital Capital Medical University Beijing China; ^2^ National Clinical Research Centre for Cardiovascular Diseases Beijing China; ^3^ Faculty of Science The University of Sydney Sydney Australia; ^4^ Heart Health Research Center Beijing China; ^5^ The George Institute for Global Health, Faculty of Medicine University of New South Wales Sydney Australia; ^6^ Liverpool Centre for Cardiovascular Science, Liverpool Heart and Chest Hospital University of Liverpool Liverpool UK

**Keywords:** atrial fibrillation, anemia, mortality, major bleeding

## Abstract

**Background:**

Anemia is a common comorbidity in patients with atrial fibrillation (AF). Reports on the association of anemia and adverse events in patients with AF, especially from Asia, are limited.

**Methods and Results:**

Based on data from the Chinese Atrial Fibrillation Registry Study (CAFR), a total of 18,106 AF patients enrolled between August 2011 and December 2018 had hemoglobin (Hb) values recorded at baseline. Patients were classified into three groups according to Hb levels: 15,606 patients (86.2%) into the no anemia group (male Hb≥130 g/L; female Hb≥120 g/L), 1800 (9.9%) with mild anemia (male 110≤Hb<129 g/L; female 110≤Hb<119 g/L), and 700 (3.9%) with moderate to severe anemia (Hb≤109 g/L). Multivariable Cox regression models were used to determine if anemia was independently associated with all‐cause death, cardiovascular death, or major bleeding, after adjusting for confounders.

Anemia was present in 13.8% of the population at baseline. During a median follow‐up of 4.01 years, the incidences of all‐cause death (1.8, 4.9, and 8.9 per 100 person‐years), cardiovascular death (1.0, 2.9, and 4.5 per 100 person‐years), and major bleeding (0.5, 0.6, and 0.7 per 100 person‐years) were gradually accentuated in patients with no anemia, mild anemia, and moderate to severe anemia, respectively. Compared with patients with no anemia, those with anemia had higher risks for all‐cause death (mild anemia; adjusted hazard ratio [HR]: 1.22, 95% confidence interval [CI]: 1.08–1.38; moderate to severe anemia; adjusted HR: 1.53, 95% CI: 1.31–1.77); and cardiovascular death (mild anemia; adjusted HR: 1.29, 95% CI: 1.10–1.52; moderate to severe anemia; adjusted HR: 1.27, 95% CI: 1.03–1.57), but not for major bleeding. The association between anemia and all‐cause death was similar among subgroups stratified by sex, kidney function, anticoagulant, or ablation therapy.

**Conclusions:**

Anemia was associated with increased risks of all‐cause death, cardiovascular death, but no major bleeding in AF patients. The effect of anemia correction on the prognosis of patients with AF requires further study.

## 1 INTRODUCTION

Anemia, defined as a decrease in hemoglobin or hematocrit, is one of the most common diseases in the world, affecting about one‐quarter of the global population.^1^ It is prevalent among women, the elderly, and people with chronic diseases (such as heart failure, diabetes mellitus, etc.),^1^ the same groups that are at high risk of atrial fibrillation (AF).

One recent large‐scale community‐based epidemiological survey conducted in China showed that the weighted AF prevalence was 1.8% among adults over 45 years old.^2^ Since the prevalence of AF and anemia increases with age, the occurrence of both is likely to be more dominant in the future as a result of an aging population. Studies have found that anemia is a common comorbidity among patients presenting with AF, but the estimated prevalence varies widely (from 12% to 37%) with most of the surveys conducted in selected AF populations.^3^, ^4^, ^5^, ^6^

Chronic anemia can induce ventricular remodeling and cardiac dysfunction^7^ with a 40% increased risk of all‐cause mortality among the general population.^8^ AF aggravates hemodynamic instability and gradually leads to changes in heart structure, which is relevant given that heart failure is one of the most important causes of death in AF.^9^ Also, compared with nonanemic AF, anemia may affect the decision whether or not to prescribe oral anticoagulant (OAC) as well as the choice of OAC.^5^ Recently, studies have shown that anemia was associated with an 80% increased risk of death in AF patients^10^ and the risk of subsequent major bleeding increased by about twofold in those on OAC.^3^, ^4^, ^5^, ^11^ However, others concluded that anemia was only associated with an increased risk of noncardiovascular death but not cardiovascular (CV) death,^12^ and not related to bleeding.^13^, ^14^

Currently, the limit of hemoglobin up to which anemia is clinically important has not been well‐investigated. Treatment of anemia in AF still lacks clear targets and specific therapy, though some anemias (such as iron deficiency) are relatively easy to correct compared with other comorbidities. For the associations of hemoglobin levels with prognosis of Asian patients in AF, data are more limited and contradictory, most are focused on the population at high risk of stroke or on OAC and had excluded patients with moderate to severe anemia (hemoglobin <10 g/dl).

Since these two diseases have overlapping effects on the CV system, we aimed to assess the associations of anemia with all‐cause mortality, CV death, and major bleeding in patients with AF by using large prospective real‐world data.

## 2 METHODS

The rationale and design of the Chinese Atrial Fibrillation Registry (CAFR) study have been previously published.^15^ In brief, this is an ongoing, prospective, multicenter, hospital‐based cohort study led by Beijing Anzhen Hospital. Thirty‐one tertiary and nontertiary hospitals in Beijing that can provide AF diagnosis and treatment services participate in this study. The CAFR registry was approved by the ethics committee of Beijing Anzhen Hospital, and all patients have signed informed consent before enrollment.

**Figure 1:**
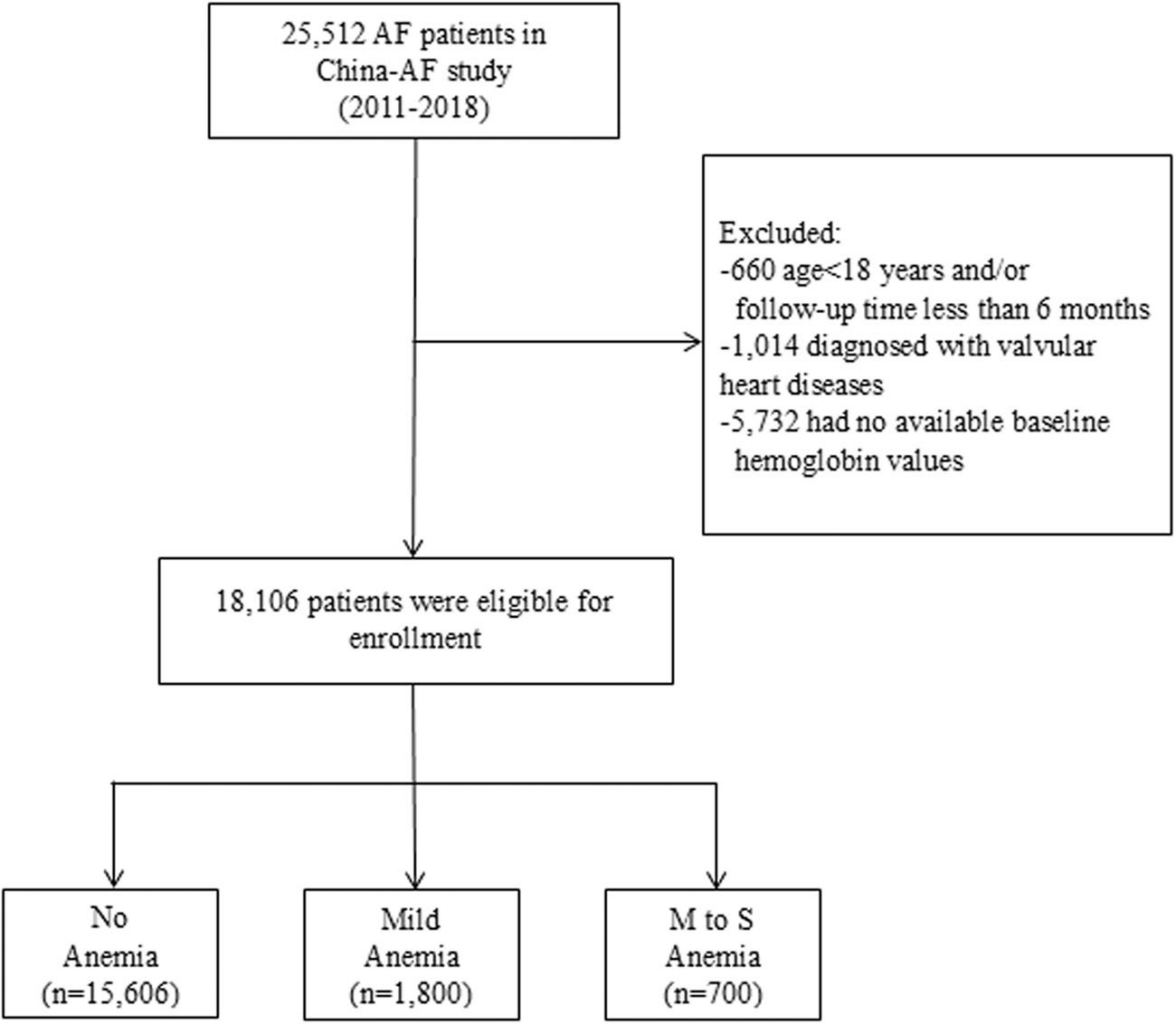
Enrollment of patients. AF, atrial fibrillation; M to S, moderate to severe.

In this study, we selected both inpatient and outpatient AF patients recruited from August 2011 to December 2018. We excluded patients if they meet any of the following: (1) age<18 years; (2) follow‐up time less than 6 months; (3) transient AF caused by reversible cause; (4) AF in the setting of concomitant valvular heart disease such as mitral stenosis, mitral valve prosthesis, and so on; and (5) missing baseline hemoglobin values. Finally, 18,106 participants were included in this analysis. Patients were divided into three groups according to their baseline hemoglobin value at the time of enrollment (see the flowchart in Figure 1).

### 2.1 Data collection

The following clinical data were collected for each patient enrolled: basic sociodemographic information (age, sex, body mass index [BMI], and level of education), combined CV risk factors (current smoking and drinking), type of AF, medical history (hypertension, chronic heart failure [CHF], established coronary artery disease [CAD], previous stroke, previous bleeding, chronic kidney disease [CKD], diabetes, etc.), CHA_2_DS_2_‐VASc score, HAS‐BLED score, results of laboratory and echocardiography tests, combined medications, and prior ablation therapy.

### 2.2 Follow‐up and clinical outcomes

Each enrolled patient was followed up in the 1st, 3rd, and 6th months, then every 6 months thereafter by trained staff under outpatient settings or through a telephone interview. Information relating to the occurrence of adverse events was collected during follow‐up. The follow‐up period was the time from enrollment to the occurrence of endpoint events or the last follow‐up time without events.

The primary endpoint was all‐cause death and the secondary endpoints were the time to CV death or major bleeding. CV deaths included deaths caused by myocardial infarction, sudden cardiac death, heart failure, and other CV diseases. Major bleeding was defined according to the International Society on Thrombosis and Haemostasis (ISTH) criteria,^16^ that is, clinically overt bleeding accompanied by transfusion of at least 2 units of whole blood or packed red cells, bleeding that required hospitalization or caused permanent dysfunction, or bleeding in important anatomical area or organ, such as intracranial, intraspinal, intraocular, retroperitoneal, pericardial, and so on, or fatal bleeding. All events were adjudicated by an independent endpoint committee.

### 2.3 Definition of anemia

Baseline hemoglobin values were derived from the latest blood test result of each patient (<1 year). The World Health Organization's (WHO) criteria of anemia^17^ were used to diagnose and classify anemia. We divided the enrolled patients into three groups: mild anemia group (110≤Hb<129 g/L for male, 110≤Hb<119 g/L for female); moderate to severe anemia group (Hb≤109 g/L); and nonanemic group (Hb130g/L or higher for male; Hb120 g/L or higher for female) according to the severity of anemia.

### 2.4 Statistical analysis

Continuous variables were presented as mean ± standard deviation (SD) or median (interquartile range [IQR]) and compared among groups using one‐way analysis of variance (ANOVA) tests or Kruskal–Wallis tests where appropriate. Categorical variables were expressed as numbers (percentages) and compared using *χ* ^2^ tests. Event rates were presented as the number of events of primary and secondary endpoints per 100 patient‐years during follow‐up. Kaplan‐Meier curves of event‐free survival of all‐cause death, CV death, or major bleeding were plotted by groups, and compared using the log‐rank tests. Step‐wised models (Model 0, Model 1, and Model 2) were performed by Cox proportional hazards regressions, adjusted for different sets of confounders, and the associations of anemia with clinical outcomes were presented with hazard ratios (HR) and their 95% confidence intervals (CIs). In Model 0, no confounder was adjusted; in Model 1, age and sex were adjusted; in Model 2, additional confounders were added to Model 1, including BMI, current smoking, education status, AF type, hypertension, CHF, CAD, stroke/transient ischemic attack/systemic embolism history, peripheral artery disease, bleeding history, CKD, diabetes, COPD, liver dysfunction, OACs, statins, antiplatelet drugs, angiotensin‐converting enzyme inhibitors + angiotensin Ⅱ receptor blockers, and ablation history. A fully adjusted Cox model (with the same confounders in Model 2) with restricted cubic splines were plotted to examine the non‐linear associations between Hb levels and all‐cause death and CV death, respectively. Knots were placed at the 25th, 50th, and 75th percentile of the distribution of Hb value. Subgroup analyses were conducted to explore the interaction effects of anemia on the risk of all‐cause death stratified by age, sex, CKD, CHF, bleeding history, OACs, and ablation therapy.

In sensitive analysis, Fine and Gray models were completed to analyze the associations between anemia severity with CV death or major bleeding, considering non‐CV death or nonmajor bleeding as competing events, respectively. In all analyses, a two‐sided *p*‐value <.05 was considered statistically significant. Statistical analyses were performed using SAS version 9.4 (SAS Institute).

## 3 RESULT

In the current analysis, we excluded 5732 patients missing baseline Hb values, and baseline characteristics of included and excluded subjects are summarized in Table S1. Finally, a total of 18,106 AF patients with available baseline Hb values were enrolled in the present analysis, of which 15,606 patients (86.2%) were free of anemia, 1800 (9.9%) were diagnosed with mild anemia, and 700 (3.9%) with moderate to severe anemia. Baseline characteristics according to the severity of anemia are shown in Table 1. In general, patients with anemia were older, more likely to be female, had lower BMI, had more comorbidities. Both CHA_2_DS_2_‐VASc and HAS‐BLED scores were significantly higher in patients with anemia. OACs prescription and ablation therapy were less prevented in anemic patients.

**Table 1:** Baseline characteristics of patients among three study groups

Patient characteristics at baseline	No anemia (*N* = 15,606)	Mild anemia (*N* = 1800)	M to S anemia (*N* = 700)	*p* value
Demographics				
Age, years	62.5 ± 11.7	70.7 ± 10.9	72.7 ± 11.9	<.001
<65	8627 (55.3)	464 (25.8)	144 (20.6)	<.001
65–74	4425 (28.4)	556 (30.9)	179 (25.6)	<.001
≥75	2554 (16.4)	780 (43.3)	377 (53.9)	<.001
Female, *n* (%)	5558 (35.6)	774 (43.0)	470 (67.1)	<.001
Personal characteristics				
BMI, kg/m²	25.7 ± 3.6	24.5 ± 3.9	24.5 ± 4.2	<.001
Normal (<24)	4307 (27.6)	722 (40.1)	286 (40.9)	<.001
Overweight (24–28)	8054 (51.6)	846 (47.0)	328 (46.9)	<.001
Obese (BMI ≥ 28)	3245 (20.8)	232 (12.9)	86 (12.3)	<.001
Current smoking, *n* (%)	2654 (17.0)	201 (11.2)	48 (6.7)	<.001
Current drinking, *n* (%)	3189 (20.4)	200 (11.1)	46 (6.6)	<.001
Highly Educated, *n* (%)	4274 (27.4)	404 (22.4)	108 (15.4)	<.001
AF type, *n* (%)				
Newly diagnosed	869 (5.6)	191 (10.6)	78 (11.1)	<.001
Paroxysmal AF	9204 (59.0)	1046(58.1)	376(53.7)	<.001
Persistent AF	5533 (35.5)	563 (31.3)	246 (35.1)	<.001
AF duration ≥1 year, *n* (%)	8984 (57.6)	983 (54.6)	365 (52.1)	0.002
Comorbidities, *n* (%)				
Hypertension	9348 (59.9)	1273 (70.7)	500 (71.4)	<.001
Chronic heart failure	1893 (12.1)	443 (24.6)	305 (43.6)	<.001
Established CAD	2185 (14.0)	424 (23.6)	185 (26.4)	<.001
Ischemic stroke/TIA/SE	2016 (12.9)	392 (21.8)	160(22.9)	<.001
Peripheral artery disease	110 (0.7)	34 (1.9)	14 (2.0)	<.001
Bleeding history	542 (3.5)	96 (5.3)	42 (6.0)	<.001
CKD	1258 (8.1)	401 (22.3)	268 (38.3)	<.001
Diabetes	3739 (24.0)	513 (28.5)	252 (36.0)	<.001
COPD	125 (0.8)	25 (1.4)	18 (2.6)	<.001
Liver dysfunction (TBIL > 34.2μmol/L or AST > 120 U/L or ALT > 165 U/L)	464 (3.0)	46 (2.6)	36 (5.1)	0.002
Hyperthyroidism/Hypothyroidism	764 (4.9)	92 (5.1)	38 (5.4)	0.766
CHA_2_DS_2_‐VASc score	2.3 ± 1.7	3.4 ± 1.9	4.2 ± 1.9	<.001
≥2, *n* (%)	9468 (60.7)	1522 (84.6)	636 (90.9)	<.001
HAS‐BLED score	1.6 ± 1.1	2.4 ± 1.3	3.4 ± 1.3	<.001
≥3, *n* (%)	3234 (20.7)	834 (46.3)	541 (77.3)	<.001
Laboratory analysis and echocardiography				
Hemoglobin(g/L)	146.7 ± 13.8	119.1 ± 5.7	97.9 ± 11.3	<.001
Heart rate (bpm)	78.9 ± 19.8	78.7 ± 21.5	80.2 ± 21.2	0.011
Left atrial diameter (mm)	40.4 ± 6.2	41.2 ± 7.4	41.8 ± 7.6	<.001
Left ventricular end diastolic dimension (mm)	48.4 ± 5.6	48.8 ± 6.4	48.2 ± 6.5	0.061
LVEF (%)	62.5 ± 8.4	61.8 ± 9.6	61.4 ± 9.7	0.019
Treatment, *n* (%)				
Antiarrhythmic drugs	6515 (41.8)	558 (31.0)	155 (22.1)	<.001
Ventricular rate control drugs	6045 (38.7)	918 (51.0)	395 (56.4)	<.001
Anticoagulant drugs	10853 (69.5)	933 (51.8)	267 (38.1)	<.001
Antiplatelet drugs	3066 (19.7)	657 (36.5)	293 (41.9)	<.001
Statins	5784 (37.1)	813 (45.2)	329 (47.0)	<.001
ACEIs/ARBs	5038 (32.3)	751 (41.7)	291 (41.6)	<.001
Ablation therapy	9764(62.6)	711 (39.5)	182 (26.0)	<.001

*Note*: CKD was defined as eGFR< 60 ml/min·1.73 m^2^ (estimated by CKD‐EPI equation).

Abbreviations: ARBs, angiotensin Ⅱ receptor blockers; ACEIs, angiotensin‐converting enzyme inhibitors; AST, aspartate amino transferase; ALT, alanine amino transferase; AF, atrial fibrillation; BMI, body mass index; CAD, coronary artery disease; COPD, chronic obstructive pulmonary disease; CKD, chronic kidney disease; LVEF, left ventricular ejection fraction; M to S, moderate to severe; SE, systemic embolism; TIA, transient ischemic attack; TBIL, total bilirubin.

### 3.1 Anemia and all‐cause death

**Figure 2:**
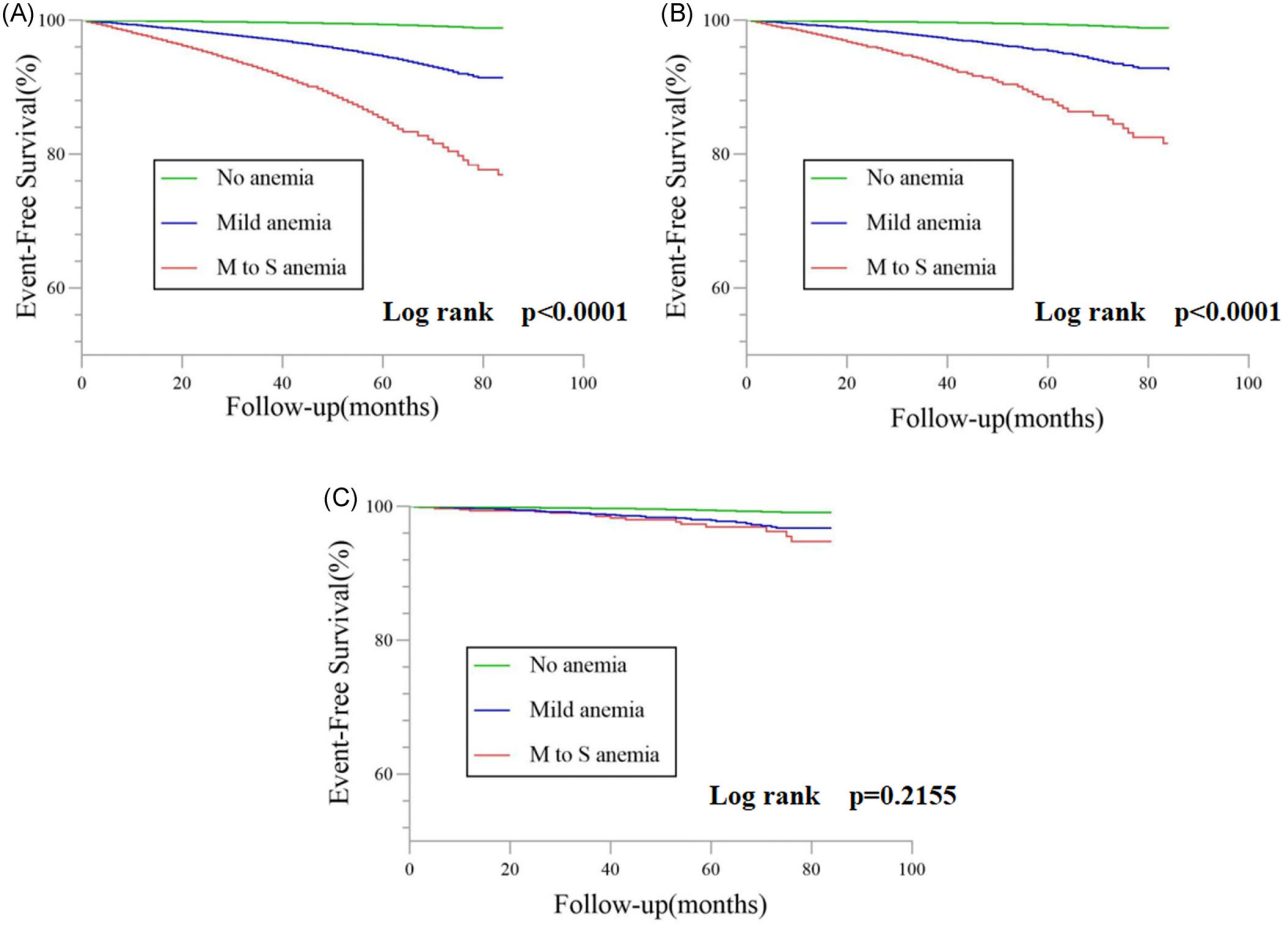
Kaplan–Meier curves for the event‐free survival among three study groups. (A) All‐cause death, (B) cardiovascular death, and (C) major bleeding. M to S, moderate to severe

The average follow‐up period was 4.01 years and a total of 1700 deaths were recorded. The incidence of all‐cause death is displayed as event‐free survival curves in Figure 2A. The crude event rates per 100 person‐years were gradually increased in the no anemia, mild anemia, and moderate to severe anemia groups. Compared with the no anemia group (1.78; 95% CI: 1.68–1.89), the incidence of all‐cause deaths in the mild anemia group increased by nearly twofold (4.86; 95% CI: 4.38–5.39, *p* < .0001), and quadrupled in the moderate to severe anemia group (8.90; 95% CI: 7.85–10.19, *p* < .0001) (Table 2).

**Table 2:** Event rates and associations of anemia with clinical outcomes

		Model 0^b^		Model 1^c^		Model 2^d^	
Endpoints	Number of events (event rate^a^)	HR (95% CI)	*p* value	HR (95% CI)	*p* value	HR (95% CI)	*p* value
All‐cause death							
No anemia	1116/1700 (1.78)	1.00 (reference)		1.00 (reference)		1.00 (reference)	
Mild anemia	358/1700 (4.86)	2.71 (2.41–3.05)	<.001	1.41 (1.25–1.60)	<.001	1.22 (1.08–1.38)	.002
M to S anemia	226/1700 (8.90)	5.07 (4.39– 5.85)	<.001	2.41 (2.08–2.80)	<.001	1.53 (1.31–1.77)	<.001
CV death							
No anemia	606/929 (0.97)	1.00 (reference)		1.00 (reference)		1.00 (reference)	
Mild anemia	210/929 (2.85)	2.90 (2.48–3.40)	<.001	1.54 (1.31–1.82)	<.001	1.29 (1.10–1.52)	.002
M to S anemia	113/929 (4.47)	4.65 (3.80–5.68)	<.001	2.21 (1.80–2.73)	<.001	1.27 (1.03–1.57)	.025
Major bleeding							
No anemia	323/389 (0.51)	1.00 (reference)		1.00 (reference)		1.00 (reference)	
Mild anemia	47/389 (0.62)	1.18 (0.87–1.60)	.297	0.90 (0.66–1.23)	.506	0.91 (0.67–1.25)	.568
M to S anemia	19/389 (0.71)	1.42 (0.89–2.25)	.141	1.00 (0.62–1.60)	.995	1.07 (0.66–1.73)	.777

Abbreviations: ARBs, angiotensin Ⅱ receptor blockers; ACEIs, angiotensin‐converting enzyme inhibitors; BMI, body mass index; CAD, coronary artery disease; CHF, chronic heart failure; CAD, coronary artery disease; COPD, chronic obstructive pulmonary disease; CKD, chronic kidney disease; CV, cardiovascular; HR, hazard ratio; CI, confidence interval; M to S, moderate to severe; OAC, oral anticoagulant; TIA, transient ischemic attack.

^a^ Event rates are presented as total number of events per 100 person‐year.

^b^ Model 0: Cox proportional hazards models without adjustment.

^c^ Model 1: Cox proportional hazards model with adjustment for age and sex.

^d^ Model 2: Model 1 with additional adjustment for BMI, current smoking, education status, AF type, hypertension, CHF, CAD, stroke /TIA/SE history, peripheral artery disease, bleeding history, CKD, diabetes, COPD, liver dysfunction, OACs, statins, antiplatelet drugs, ACEIs+ARBs and ablation therapy.

**Figure 3:**
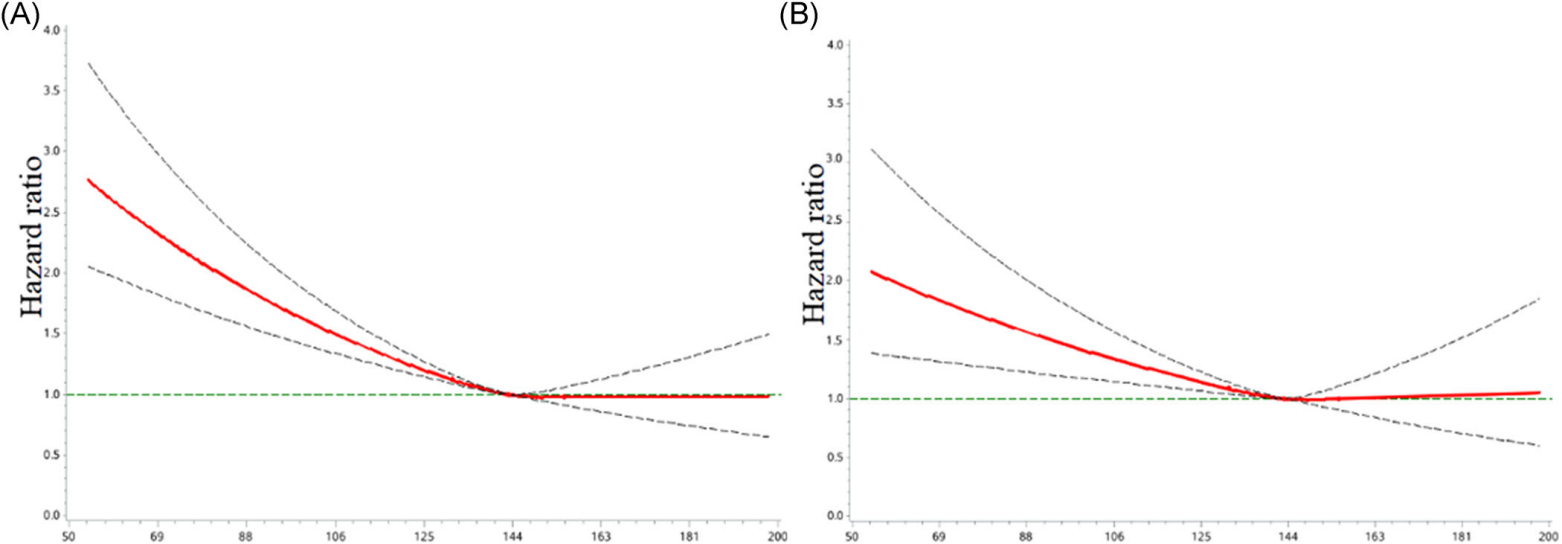
Graphic representation of the HR (95% CI) for all‐cause death according to baseline hemoglobin levels. Adjusted for age, sex, BMI, current smoking, education status, AF type, hypertension, CHF, CAD, stroke/TIA/SE history, peripheral artery disease, bleeding history, CKD, diabetes, COPD, liver dysfunction, OACs, statins, antiplatelet drugs, ACEIs+ARBs, and ablation therapy. ARBs, angiotensin Ⅱ receptor blockers; ACEIs, angiotensin‐converting enzyme inhibitors; AF, atrial fibrillation; CAD, coronary artery disease; CHF, chronic heart failure; CAD, coronary artery disease; COPD, chronic obstructive pulmonary disease; TIA, transient ischemic attack

On Multivariate Cox regression analysis from the fully adjusted model (Model 2), baseline anemia was significantly related to increased risk for all‐cause death (mild; adjusted HR: 1.22, 95% CI: 1.08–1.38; moderate to severe; adjusted HR: 1.53, 95% CI: 1.31–1.77) in AF patients, compared with those without anemia (Table 2). Notably, a dose‐dependent relationship of anemia with all‐cause death was observed. When Hb was considered as a continuous variable, as shown in Figure 3A, decreasing Hb was associated with an increased risk of all‐cause death. The cutoff value was 143 g/L.

Subgroup analyses stratified separately by age, sex, CHF, CKD, OACs, and ablation therapy were conducted across different groups (Table S2). Interaction analysis revealed a significant interaction with age for the all‐cause death. Though anemia was associated with worsening outcomes in both age groups, the association was consistently stronger in younger patients (age < 65). In the moderate to severe anemia group, patients without CHF had higher risks of all‐cause death compared with CHF patients, while no difference between subgroups was found in the mild anemia group. The association between anemia and the all‐cause death did not differ according to sex, kidney function, and treatment allocation (*p* for interaction >.05, Table S2).

### 3.2 Anemia and CV death

During the follow‐up period, a total of 929 CV deaths were recorded. A significant association was also observed between anemia and CV death. When compared with no anemia, mild anemia (adjusted HR: 1.29, 95% CI: 1.10–1.52), and moderate to severe anemia (adjusted HR: 1.27, 95% CI: 1.03–1.57) were significantly associated with a higher risk of CV death (Table 2). The same findings were observed in the analyses using Hb as a continuous variable (Figure 3B).

### 3.3 Anemia and major bleeding

A total of 389 major bleeding events occurred. The event rates and 95% CIs were 0.51 (95% CI: 0.46–0.57), 0.62(95% CI: 0.47–0.83), 0.71(95% CI: 0.46–1.12) per 100 person‐years for the no anemia group, mild anemia group, and moderate to severe anemia group, respectively (Table 2). Upon both univariate and multivariate analysis, there were no significant statistical correlations between anemia and major bleeding in subjects (both *p*‐value＞.1; Table 2).

### 3.4 Sensitivity analyses

When death was considered a competing risk, the results remained unchanged. Lower hemoglobin was still associated with a significantly higher risk of CV death. The association between anemic status and major bleeding also remained statistically insignificant (Table S3).

## 4 DISCUSSION

Our principal findings in this analysis from a contemporary nationwide AF registry are summarized as follows: (i) anemia was present in 13.8% of the studied population, which indicates anemia as common comorbidity in AF patients; and (ii) anemia is associated with a significant increase in the adjusted risk of both all‐cause death and CV death wherein progressive degrees of anemia portended worse outcomes, but not for major bleeding in AF patients.

### 4.1 Anemia and death

Baseline hemoglobin level or anemia is associated with a 41% increase in all‐cause mortality among the general population^8^ while a 78% increase in AF population,^10^ which indicate anemia may exacerbate AF progression through chronic cardiac remodeling. In our analysis, baseline anemia was statistically associated with both all‐cause death and CV death in AF patients in which lower hemoglobin is strongly related to poor survival outcomes. Interestingly, we were able to demonstrate a stronger association in the younger age group compared with older patients for all‐cause death. This conclusion is consistent with results in post‐hoc analysis of RE‐LY^4^ and ARISTOTLE^3^ trials.

Currently, we are unable to explain the etiology of this interaction, but we speculate that younger patients potentially experienced prolonged severity and duration of anemia, while anemia is more physiological in older patients. Also, normal Hb distribution varies not only with sex but also with ethnicity and physiological status. Although there is still no uniform standard, new lower limits of normal Hb values have been proposed according to age.^18^ Thus, normality ranges of Hb may be interpreted differently in the young and elderly.

There are pathophysiological explanations to the hypothesis that the presence of anemia could directly lead to worse outcomes among AF patients. In a perennial anemic state, hypoxemia and hypoperfusion trigger an increase in heart rate and stroke volume to increase cardiac output in a reflex response, leading to cardiac remodeling. Dilation of the left chambers secondary to anemia results in increased systolic wall stress and compensatory hypertrophy.^19^ Indeed, anemia may be an independent predictor of subclinical myocardial damage, after adjusting for confounders.^20^ In addition, anemia has been suggested to be related to the inflammation response,^21^ which could be involved in affecting the prognosis of AF through inflammation‐induced alteration of electrophysiological properties, remodeling of cardiac structure, and enhancement of fibrosis.^22^ All these factors could gradually lead to heart failure and potentially worsen myocardial oxygen supply.

### 4.2 Anemia and major bleeding

The nonvitamin K antagonist oral anticoagulants (NOACs) are now widely for the prevention of AF‐related stroke^23^ but all OACs come at the cost of increased bleeding risk.^24^ In patients with AF, anemia has primarily been considered as a predictor of bleeding.^25^, ^26^, ^27^ When assessing the consistency of the association of anemia and bleeding risk, contrary to expectations, the association between anemia and major bleeding in this study was not significant. Although post hoc analyses of randomized controlled trials (RCTs) have indicated that anemia is associated with increased risk of subsequent major bleeding by about twofold in AF patients who were on OAC therapies,^3^, ^4^, ^11^ others have shown negative results.^13^, ^14^, ^28^

Indeed, one analysis from a prospective study in the United Kingdom concluded that anemia did not appear to be a predictive factor for bleeding.^13^ In parallel, two studies also indicated that hemoglobin concentration was not related to the occurrence of major bleeding after adjusting for variables.^14^, ^28^ Several possible explanations may account for why anemia was not associated with increased bleeding risk in our population. First, the association between anemia and bleeding is vulnerable to reverse causality in which lower hemoglobin might be a result rather than a cause of bleeding. Reverse causality exists in other studies. For example, AF patients with OAC treatment were more likely to be diagnosed with pre‐existing cancer.^29^, ^30^ In this scenario, OACs could be considered as a “bleeding stress test,” which therefore could potentially unveil occult cancer by triggering a bleeding episode and improve the chance of early detection. In previous studies, when evaluating the relationship between anemia and bleeding in patients with AF, few efforts have been taken to reduce this potential bias. Second, the anticoagulation rate is different in various studies, perhaps contributing to conflicting results. The low proportion of effective anticoagulation in patients with AF has long been a problem, especially in the Asian areas. Meanwhile, the 1‐year and 2‐year withdrawal rates of prescribed anticoagulants in AF patients have been as high as 44% and 58%, respectively.^31^ We speculate poor patient compliance and suboptimal quality of anticoagulation control could conceivably have a significant impact on bleeding.

### 4.3 Clinical relevance

In essence, anemia is an independent risk factor for all‐cause death and CV death in patients with AF. Anemia correction such as iron therapy could favorably affect outcomes in patients with other CV diseases after diagnosis of anemia; for example, in iron‐deficient heart failure patients, treatment with ferric carboxymaltose was associated with a lower rate of total heart failure hospitalizations or CV mortality.^32^ Cardiac geometry also changed significantly in echocardiography after appropriate correction of the low hemoglobin levels in anemia patients, which might improve cardiac structure and function.^33^ For now, no such trials have been conducted in people with AF.

Many studies have reported that the mortality of AF patients remains high even after OAC treatment,^34^ indicating interventions beyond anticoagulation are needed to further reduce mortality in AF. Clinicians should evaluate the patient's general condition and conduct comprehensive management of other comorbidities, as proposed in the integrated Atrial fibrillation Better Care (ABC) pathway^35^ and recommended in the 2020 European Society of Cardiology Guidelines. Optimal management with ABC pathway adherence has been associated with improved clinical outcomes.^36^, ^37^, ^38^ Unfortunately, current guidelines fail to recommend a specific anticoagulation strategy or an exact critical value of hemoglobin for AF patients with anemia. Treatment of anemia in patients with AF still lacks clear targets and specific therapy has not developed in guidelines. The addition of this aspect in the new guidelines is to expect. Clinically, anemia may affect the decision‐making of clinicians, and patients with anemia are frequently not prescribed with OACs presumably out of bleeding concerns.^5^ This is also reflected in our observations. Many anemia patients are asymptomatic until advanced stages of the disease. Proactive screening of anemia could potentially provide early detection of underlying disease and could result in earlier treatment, better prognosis, and possibly increased long‐term survival.

### 4.4 Strength and limitations

The strength of the current analysis is the novelty of the population studied, that is, a large, well‐defined data set and the careful adjustment for potential confounders. As far as we know, this is the first time anemia has been analyzed in the Chinese AF population. There are still several inevitable limitations in our current study. First, we only collected baseline hemoglobin values but did not record them in the subsequent follow‐up periods. Unfortunately, we fail to observe how dynamic changes in hemoglobin will affect these specific endpoints. Second, data on the cause of anemia were unavailable in this analysis. Special conditions such as CKD or recent bleeding could have potential effects on Hb concentration, although CKD and bleeding history still did not differ in the subgroup analysis of all‐cause deaths. Third, 5732 patients were excluded from this study population due to the lack of baseline Hb data, which could result in a selection bias. Fourth, we did not have an age‐matched control cohort of Chinese people without AF that would have allowed us to compare the prognostic impacts of anemia in patients with and without AF. Finally, all the results were from a prospective observational study. Despite adjustments for clinically relevant factors through multivariate analysis, it still shows the inherent limitations of the study design (such as selection bias and unmeasurable confounding factors). Also, a reverse causation effect may not be fully eliminated. Further studies are needed to further verify whether anemia correction could favorably affect outcomes in AF patients with anemia.

## 5 CONCLUSION

Anemia was associated with increased risks of all‐cause death, CV death, but no major bleeding in AF patients. The effect of anemia correction on the prognosis of patients with AF requires further study.

## CONFLICT OF INTERESTS

Chang‐Sheng Ma has received honoraria from Bristol‐Myers Squibb, Pfizer, Johnson & Johnson, Boehringer‐Ingelheim, and Bayer for giving lectures. Jian‐Zeng Dong has received honoraria from Johnson & Johnson for giving lectures. The remaining authors declare that there are no conflict of interests.

## Supporting information

Supporting information.

## Data Availability

The data that support the findings of this study are available on request from the corresponding author. The data are not publicly available due to privacy or ethical restrictions.
