# Supplementary material for: Associations of anemia with death and major bleeding in patients with atrial fibrillation: A report from the Chinese Atrial Fibrillation Registry Study
Source: Clin Cardiol. 2021 Dec 28;45(1):91–100. doi: 10.1002/clc.23764 (PMC8799039; doi:10.1002/clc.23764)
Supplement: Supplementary file 1 — Supporting information. [file CLC-45-91-s001.docx]

**SUPPLEMENTAL MATERIAL**

**TableS1** Baseline clinical characteristics of Included and Excluded Participants

| Patient Characteristics at Baseline | | Included  (n=18106) | Excluded  (n=5732) | P  value |
| --- | --- | --- | --- | --- |
| Demographics | Age, yrs | 63.7±12.0 | 64.5±12.0 | <.0001 |
|  | ＜65 | 9235(51.0) | 2705 (47.2) | <.0001 |
|  | 65-74 | 5160 (28.5) | 1772 (30.9) | <.0001 |
|  | ≥75 | 3711 (20.5) | 1255 (21.9) | <.0001 |
|  | Female, n (%) | 6802 (37.6) | 2229 (38.9) | 0.0727 |
| Personal  Characteristics | BMI, kg/m² | 25.6±3.7 | 25.4±3.7 | 0.0066 |
|  | Normal (<24) | 5314(29.3) | 1756 (30.6) | 0.0064 |
|  | Overweight (24–28) | 9228(51.0) | 2952 (51.5) |  |
|  | Obese (BMI ≥28) | 3563 (19.7) | 1024 (17.9) |  |
|  | Current Smoking, n (%) | 2903 (16.0) | 756 (13.2) | <.0001 |
|  | Current Drinking, n (%) | 3435 (19.0) | 959 (16.7) | 0.0001 |
|  | Highly Educated, n (%) | 4786(26.4) | 1798 (31.4) | <.0001 |
| AF type | AF type, n (%) |  |  |  |
|  | Newly diagnosed | 1138 (6.3) | 190 (3.3) | <.0001 |
|  | Paroxysmal AF | 10626(58.7) | 3430(59.9) | <.0001 |
|  | Persistent AF | 6342 (35.0) | 2112 (36.9) | <.0001 |
|  | AF duration≥ 1 year, n (%) | 10332 (57.1) | 3174 (55.4) | 0.0244 |
| Comorbidities, n (%) | Hypertension | 11121 (61.4) | 3396 (59.3) | 0.0033 |
|  | Chronic heart failure | 2641 (14.6) | 488 (8.51) | <.0001 |
|  | Established CAD | 2794 (15.4) | 681 (11.9) | <.0001 |
|  | Ischaemic stroke/TIA/SE | 2568 (14.2) | 825 (14.4) | 0.6920 |
|  | Peripheral artery disease | 158 (0.9) | 17 (0.3) | <.0001 |
|  | Bleeding History | 680 (3.8) | 217 (3.8) | 0.9169 |
|  | CKD | 1927 (10.6) | 184 (3.2) | <.0001 |
|  | Diabetes | 4504 (24.9) | 973 (17.0) | <.0001 |
|  | COPD | 168 (0.9) | 35 (0.6) | 0.0227 |
|  | Liver dysfunction (TBIL>34.2μmol/L or AST>120U/L or ALT>165 U/L) | 546 (3.0) | 44 (0.8) | <.0001 |
|  | Hyperthyroidism/Hypothyroidism | 894 (4.9) | 284 (5.0) | 0.9586 |
| CHA2DS2-VASc score | | 2.5±1.8 | 2.3±1.7 | 0.0059 |
| ≥2, n (%) | | 11626 (64.2) | 3670 (64.0) | 0.7999 |
| HASBLED score | | 1.8±1.2 | 1.7±1.0 | 0.3566 |
| ≥3, n (%) | | 4609 (25.5) | 1295 (22.6) | <.0001 |
| Laboratory analysis and Echocardiography | Hemoglobin(g/L) | 142.0 ±17.9 | - | - |
|  | Heart Rate (bpm) | 78.9±20.0 | 78.9±17.3 | 0.2288 |
|  | Left atrial diameter (mm) | 40.5±6.4 | 40.3±6.8 | <.0001 |
|  | Left ventricular end diastolic dimension (mm) | 48.5±5.7 | 48.2±5.6 | 0.0436 |
|  | LVEF (%) | 62.4±8.6 | 62.4±8.5 | 0.5130 |
| Treatment, n(%) | Antiarrhythmic drugs | 6515 (41.8) | 558 (31.0) | <.0001 |
|  | Ventricular rate control drugs | 7358 (40.6) | 2732 (47.7) | <.0001 |
|  | Anticoagulant drugs | 12053(66.6) | 2595 (45.3) | <.0001 |
|  | Antiplatelet drugs | 4016(22.2) | 1535 (26.8) | <.0001 |
|  | Statins | 6926 (38.3) | 1466 (25.6) | <.0001 |
|  | ACEI/ ARB | 6080 (33.6) | 1548 (27.0) | <.0001 |
|  | RFCA | 10657(58.9) | 1141 (19.9) | <.0001 |

BMI, body mass index; AF, atrial fibrillation; CAD, coronary artery disease; TIA, transient ischemic attack; SE, systemic embolism; LVEF, left ventricular ejection fraction; COPD, chronic obstructive pulmonary disease; TBIL, total bilirubin; AST, aspartate amino transferase; ALT, alanine amino transferase;CKD，chronic kidney disease；CKD was defined as eGFR< 60 ml/min·1.73m^2^(estimated by CKD-EPI equation); ACEIs, angiotensin-converting enzyme inhibitors; ARBs, angiotensin Ⅱ receptor blockers; RFCA, radiofrequency catheter ablation.

**TableS2** Subgroup analysis for all-cause death.

| Subgroups | | ad HR*(95% CI) | P value | P  for interaction |
| --- | --- | --- | --- | --- |
| **Age ≥65**  Mild Anemia  M to S Anemia | No  Yes  No  Yes | 2.10 (1.43-3.06)  1.14 (1.00-1.30)  2.43 (1.32-4.45)  1.47 (1.26-1.72) | 0.002  0.043  0.004  <.001 | 0.001  0.041 |
| **Female**  Mild Anemia  M to S Anemia | No  Yes  No  Yes | 1.28 (1.09-1.50)  1.17 (0.95-1.43)  1.68 (1.34-2.10)  1.43 (1.16-1.76) | 0.003  0.136  <.001  0.001 | 0.557  0.311 |
| **CHF**  Mild Anemia  M to S Anemia | No  Yes  No  Yes | 1.21 (1.01-1.45)  1.21 (1.02-1.44)  1.92 (1.50-2.46)  1.37 (1.13-1.66) | 0.036  0.028  <.000  0.002 | 0.592  0.005 |
| **CKD**  Mild Anemia  M to S Anemia | No  Yes  No  Yes | 1.20 (1.02-1.40)  1.22 (1.00-1.50)  1.46 (1.17-1.81)  1.57 (1.26-1.96) | 0.024  0.052  0.001  <.001 | 0.673  0.882 |
| **Bleeding History**  Mild Anemia  M to S Anemia | No  Yes  No  Yes | 1.25 (1.10-1.42)  0.98 (0.60-1.60)  1.56 (1.33-1.83)  1.22 (0.64-2.32) | 0.006  0.932  <.001  0.556 | 0.153  0.266 |
| **OAC**  Mild Anemia  M to S Anemia | No  Yes  No  Yes | 1.27 (1.09-1.46)  1.12 (0.88-1.43)    1.52 (1.28-1.80)  1.51 (1.08-2.12) | 0.002  0.345  <.001  0.017 | 0.402  0.955 |
| **Ablation Therapy**  Mild Anemia  M to S Anemia | No  Yes  No  Yes | 1.24 (1.09-1.41)  1.16 (0.79-1.71)  1.50 (1.28-1.76)  2.09 (1.19-3.65) | 0.002  0.440  <.001  0.010 | 0.842  0.264 |

HR: hazard ratio; CI: confidence interval; M to S, moderate to severe.

*Adjusted for age, sex, BMI, current smoking, education status, AF type, hypertension, CHF, CAD, stroke /TIA/SE history, peripheral artery disease, bleeding history, CKD, diabetes, COPD, liver dysfunction, OACs, statins, antiplatelet drugs, ACEIs+ARBs and ablation therapy. See Table 2 footnote for expansion of abbreviation.

**TableS3** Association between anemia and endpoints in 3 study groups in Fine and Gray models.

| Endpoints | Model 0^†^ | | Model 1^‡^ | | Model 2^§^ | |
| --- | --- | --- | --- | --- | --- | --- |
|  | HR  (95% CI) | P value | HR  (95% CI) | P value | HR  (95% CI) | P  value |
| **CV death** |  |  |  |  |  |  |
| No anemia | 1.00(reference) |  | 1.00(reference) |  | 1.00(reference) |  |
| Mild anemia | 2.82(2.41-3.31) | <.001 | 1.55(1.31-1.84) | <.001 | 1.29(1.09-1.53) | 0.003 |
| M to S  anemia | 4.19(3.42-5.13) | <.001 | 1.96(1.58-2.45) | <.001 | 1.27(1.02-1.58) | 0.030 |
| **Major Bleeding** |  |  |  |  |  |  |
| No anemia | 1.00(reference) |  | 1.00(reference) |  | 1.00(reference) |  |
| Mild anemia | 1.10(0.81-1.50) | 0.533 | 0.88(0.64-1.20) | 0.408 | 0.91(0.66-1.25) | 0.569 |
| M to S  anemia | 1.18(0.74-1.87) | 0.489 | 0.85(0.52-1.39) | 0.518 | 1.07(0.65-1.76) | 0.785 |

CV: cardiovascular; HR: hazard ratio; CI: confidence interval; M to S: moderate to severe.

†Model 0: Cox proportional hazards models without adjustment.

‡Model 1: Cox proportional hazards model with adjustment for age and sex.

§Model 2: Model 1 with additional adjustment for BMI, current smoking, education status, AF type, hypertension, CHF, CAD, stroke /TIA/SE history, peripheral artery disease, bleeding history, CKD, diabetes, COPD, liver dysfunction, OACs, statins, antiplatelet drugs, ACEIs+ARBs and ablation therapy.
